# Application of a bioinformatics training delivery method for reaching dispersed and distant trainees

**DOI:** 10.1371/journal.pcbi.1008715

**Published:** 2021-03-18

**Authors:** Christina R. Hall, Philippa C. Griffin, Andrew J. Lonie, Jeffrey H. Christiansen

**Affiliations:** 1 Australian BioCommons, Australia; 2 EMBL Australia Bioinformatics Resource, Australia; 3 Melbourne Bioinformatics, University of Melbourne, Victoria, Australia; 4 Research Computing Centre, The University of Queensland, Queensland, Australia; 5 Queensland Cyber Infrastructure Foundation, Queensland, Australia; SIB Swiss Institute of Bioinformatics, SWITZERLAND

## Abstract

Many initiatives have addressed the global need to upskill biologists in bioinformatics tools and techniques. Australia is not unique in its requirement for such training, but due to its large size and relatively small and geographically dispersed population, Australia faces specific challenges. A combined training approach was implemented by the authors to overcome these challenges. The “hybrid” method combines guidance from experienced trainers with the benefits of both webinar-style delivery and concurrent face-to-face hands-on practical exercises in classrooms. Since 2017, the hybrid method has been used to conduct 9 hands-on bioinformatics training sessions at international scale in which over 800 researchers have been trained in diverse topics on a range of software platforms. The method has become a key tool to ensure scalable and more equitable delivery of short-course bioinformatics training across Australia and can be easily adapted to other locations, topics, or settings.

## Introduction

Over recent years, significant technological advances and lowering costs of biological molecule sensing technologies have led to the routine generation of data at large scale in the life sciences, and biological research has been greatly supported by the adoption of data science approaches. The global adoption of these methodologies is reflected by the increase of data stored in international repositories such as the ELIXIR Core Data Resources [1] and those managed by the National Center for Biotechnology Information [2].

The ever-increasing requirement for biologists to wrangle large quantities of complex data presents a rolling skills deficit in a fast-changing research environment. The full potential of biological data will be realised when life science researchers can skilfully manipulate and analyse data at large scale [3]. Researchers perceive a need for training to support better use of a variety of data-related or computational components that underpin their research [4]. Since research groups often lack team members with bioinformatics experience and skills, self-learning practices may result in “reinventing the wheel” along with an increased risk of the adoption of bad practices [5]. The challenge of continuous upskilling to process and analyse biological data is felt globally, and many initiatives of varied scope and scale around the world offer bioinformatics training programs for researchers (S1 Table).

Australia is no exception in having a critical need to upskill biologists to handle biological data at scale [6,7], and a number of bioinformatics training programs and resources that are intended for national, state, or institutional audiences have been developed (S2 Table). A significant past national effort focussed on developing a network of bioinformatics trainers who travelled to deliver hands-on bioinformatics workshops around the country [8]. While this initiative was active, 19 professional Australian bioinformaticians participated in EMBL-EBI’s Train-the-Trainer program [7] and trained over 1,300 participants in 47 face-to-face training events between 2012 and 2017 (A. Gilbert, Bioplatforms Australia, pers. comm.) using a purpose-built computational environment and consistent training materials [8,9].

Shortfalls in the abilities of Australian life scientists to confidently apply bioinformatics approaches to their research persist. Excellent open-access repositories of high-quality training materials are available online (e.g., TeSS [https://tess.elixir-europe.org/], Galaxy Training Network [https://training.galaxyproject.org/training-material/], and GOBLET [https://mygoblet.org/training-materials/]), but access to skilled trainers remains a challenge. Researchers requiring training are often dispersed and isolated from each other. Australia is a large country (roughly comparable in size to the continental United States of America at 7.69 million km^2^; https://www.ga.gov.au/scientific-topics/national-location-information/dimensions) with a relatively small population (25.6 million on March 2020, i.e., less than half the population of a large European country like France or Italy; https://www.abs.gov.au/statistics/people/population) that is densely concentrated in geographically distant urban areas. Moreover, 1 to 2 hours flying time is required to travel between every pair of the nearest state capitals. Regional university campuses may also be located several hours drive away from their own university’s main campus.

### Distributed national research infrastructure providing bioinformatics support to Australian life science researchers

The EMBL Australia Bioinformatics Resource (EMBL-ABR) worked to maximise Australia’s bioinformatics capability from 2016 to 2019. With Bioplatforms Australia and University of Melbourne funding, this collaboration with EMBL-EBI established a national network of 13 nodes representing institutions supporting areas of relevance to bioinformatics—including training [10].

National training and other activities undertaken within EMBL-ABR have been pivotal in the formation of the Australian BioCommons (established July 2019; https://www.biocommons.org.au/). Australian BioCommons is streamlining Australian research communities’ access and usage of digital research analysis resources that are developed in concert with international peer infrastructures. In its mission to facilitate access to the digital techniques required for world-class bioscience, Australian BioCommons has continued to hone a “hybrid” training technique implemented by EMBL-ABR. Events delivered in this format complement a broad training program which includes other delivery formats. Analysis of content design, competency frameworks, and long-term impact of the broader Australian BioCommons training program is outside of the scope of this article. In this paper, the key characteristics of the hybrid training delivery method are described, along with practical checklists for those wanting to set up similar training events.

### “Hybrid” training: Accessible, scalable bioinformatics training

While the hybrid method as described here has grown out of the unique needs of Australian life science researchers seeking training in bioinformatics skills, components of the delivery model have been influenced by other activities such as the work of the H3ABioNet Consortium to combine online bioinformatics training delivery with face-to-face support [11,12] and The Carpentries workshops (https://carpentries.org).

The “hybrid” method’s first incarnation evolved to deliver training after a national roadshow-style tour by an international expert was cancelled at the last minute. To connect this USA-based expert with dispersed Australian researchers, the workshop went ahead in an online format after intensive preliminary training for local facilitators. The ability for facilitators and participants to gain direct access to the expertise regardless of their location was compelling.

Live distance training via webinars provides “low cost, short duration, flexible, and potentially global access” [13]; however, the interactivity and engagement of a webinar format cannot compete with longer format face-to-face training. The hybrid training method combines online presentations delivered live by an expert trainer, with practical tutorials in a classroom setting that are supported by local facilitators. It supports the efficient and scalable provision of live, hands-on training events across a dispersed network with the ability for all participants to interact remotely, in real time with the trainer/s while under the direct supervision by in-room pretrained facilitators.

**Fig 1:**
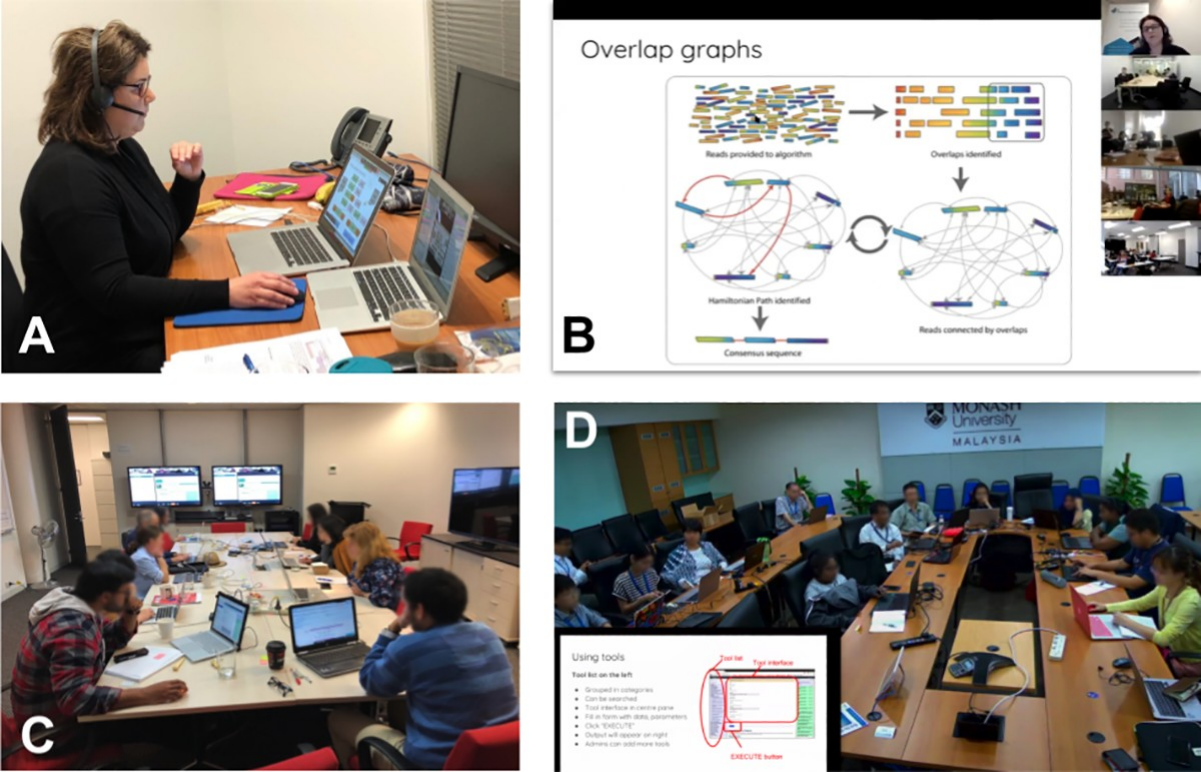
A hybrid training event underway. (A) The trainer sits separate to the audience to maintain focus on delivering the training. (B) Each venue views the trainer’s presentation as well as the camera views of the trainer and other participating sites. (C) A venue in Melbourne, Australia shows a small group of participants viewing the presentation on communal screens while individually working through exercises. (D) The ability for the convenor and trainer to view venues, such as this snapshot of a large group in Malaysia, provides instant feedback on participants’ progress.

Participants at multiple venues simultaneously watch live presentations from an expert trainer and work through interactive practical exercises with face-to-face guidance from the trained facilitators (Fig 1). Many opportunities exist for participants to ask questions during scheduled question times, interactive group work, peer discussions, or via a shared editable “discussion board.” Events of this type have successfully trained up to 120 participants in simultaneous workshops around Australia and beyond (see Table 1).

**Table 1:** Bioinformatics training events using the hybrid methodology.

Workshop	Trainer location	No. of locations/facilitators	Training platform and materials used	No. of participants per city	Web views (November 2020)
Genome Annotation using Apollo (November 2017)	San Francisco Bay Area, USA	8/15	Virtual machines running Apollo instances hosted on Nectar Openstack cloud Material provided by Apollo project	Brisbane: 16 Townsville: 5 Cairns: 7 Melbourne: 28 Sydney:14 Hobart: 5 Adelaide: 18 Total: 93	884
Introduction to Galaxy Australia (I): Genome Assembly and Annotation (August 2018)	Melbourne, Australia	9/11	Galaxy Australia managed service Galaxy Australia/Melbourne Bioinformatics Training Material	Brisbane: 11 Toowoomba: 6 Cairns: 4 Townsville: 11 Sydney: 2 Melbourne: 21 Adelaide: 26 Hobart: 13 Total: 94	3,318
Introduction to Galaxy Australia (II): Finding genetic variants in bacterial sequence data (September 2018)	Brisbane, Australia	11/16	Galaxy Australia managed service Galaxy Australia/Melbourne Bioinformatics Training Material	Brisbane: 3 Toowoomba: 6 Cairns: 4 Townsville: 1 Sydney: 2 Melbourne: 21 Adelaide: 9 Hobart: 10 Kuala Lumpur: 16 Total: 72	330
Introduction to Galaxy Australia (III): Differential Gene Expression from Bacterial RNA-seq Data (October 2018)	Melbourne, Australia	11/17	Galaxy Australia managed service Galaxy Australia/Melbourne Bioinformatics Training Material	Brisbane: 4 Toowoomba: 5 Cairns: 3 Townsville: 4 Sydney: 5 Melbourne: 21 Adelaide: 16 Hobart: 8 Kuala Lumpur: 14 Total: 82	1,829
Metagenomics (16S) using Galaxy Australia (November 2018)	Melbourne, Australia	11/17	Galaxy Australia managed service Galaxy Training Network/Melbourne Bioinformatics	Brisbane: 9 Toowoomba: 5 Cairns: 2 Townsville: 3 Sydney: 8 Melbourne: 10 Adelaide: 6 Hobart: 5 Kuala Lumpur: 11 Total: 59	1,768
UCSC Genome Browser: a full-featured genomic data system (November 2018)	Santa Cruz, USA	5/9	UCSC Public Genome Browser Material developed by UCSC Project	Brisbane: 8 Townsville: 7 Sydney: 8 Melbourne: 10 Adelaide: 10 Total: 43	292
Introduction to Snakemake and Nextflow (June 2019)	Adelaide, Australia	8/18	Virtual SLURM cluster in AWS Materials developed by trainers from University of Adelaide and CSIRO	Brisbane: 14 Canberra: 18 Townsville: 5 Sydney: 20 Melbourne: 29 Adelaide: 21 Perth: 14 Total: 121	n/a
Phylogenetics—Back to Basics (November 2019)	Hobart, Australia	9/11	Galaxy Australia managed service and Splitstree software installed on participant laptops Materials developed by trainers from University of Tasmania	Brisbane: 17 Toowoomba: 15 Townsville: 10 Sydney: 30 Melbourne: 44 Adelaide: 9 Hobart: 7 Perth: 28 Total: 120	2,235
Using Circos in Galaxy Australia (February 2020)	Melbourne, Australia	7/12	Galaxy Australia managed service Materials developed by trainer with reference to Galaxy Training Network materials	Brisbane: 20 Toowoomba: 6 Hobart: 9 Melbourne: 41 Canberra: 7 Perth: 11 Total: 94	523

Nine training events were held between 2017 and 2020, bringing trainers from the USA and 4 Australian states together with 808 workshop participants joining from multiple venues in Australia and Malaysia. For each event, each city listed had 1 participating site with the exception of Melbourne, which had either 2 or 3 sites.

### Features of the hybrid method for bioinformatics training

#### Trainers present online

Each training event is coordinated by a convenor and led by 1 or more experienced and expert trainers. The trainer works closely with the convenor to create a 3- to 4-hour highly structured program. The session includes webinar-style presentations delivered live by the lead trainer and active hands-on exercises to engage the remote audience. During the event broadcast, the trainer is accompanied by an off-screen assistant for support with any problems at venues or questions that require immediate action. The trainer and facilitators address participants’ questions at designated times via a collaborative online “discussion board” shared document or as they arise in rooms. Facilitators are trained ahead of time by the trainer and often contribute to the development of the training materials. Their major role is to host local participants in the classroom: to guide them through the materials, demonstrate effective use of the computational environment, and lead the hands-on exercises while ensuring their group progresses in sync with other venues. As well as providing critical face-to-face guidance, facilitators encourage local networking, drive interactions across venues, and answer questions on the discussion board.

#### Local venues with a shared screen displaying presentation

Participants gather with members of their research community at a local venue. Learning in a classroom setting facilitates networking among participants, encourages shared troubleshooting, enables relationship building with peers, offers guidance from trained facilitators, and engenders a feeling of community.

#### Planning requirements

Access to a computer is required, with participants preferably continuing to use the same device once the training is complete.

Trainers develop the content ensuring it is fit for purpose for the hybrid method and train facilitators with the assistance of the convenor. They select, test, and resource an appropriate computational platform for participants’ use. Access to a good quality microphone, camera, and internet connection and an assistant are essential.

The availability of training at particular locations is dependent on facilitator availability, with an approximate ratio of 1:10 facilitators to participants strongly encouraged. Facilitators manage the local logistics and work with the convenor to ensure venues with good quality cameras, screens, and internet connectivity are available.

Facilitator training ideally involves collaborative generation of the training materials which may require a face-to-face meeting before the workshop. Links to join the event online are only provided to facilitators to ensure group attendance. The centralised coordination by the convenor includes planning, advertising, registrations, communications, evaluations, and reporting. The recording of the event and perpetual documents are provided online for participants’ prolonged use.

A detailed checklist for planning and undertaking a hybrid training event is provided (S1 Checklist). Australian BioCommons manages registrations by Eventbrite (https://www.eventbrite.com.au/) and hosts events via Zoom (https://zoom.us/). Discussion boards and agendas were created in Google Docs (https://docs.google.com/). The agenda listed links to all resources, according to the preference of the lead trainer (e.g., https://tinyurl.com/galaxyworkshop3 and https://tinyurl.com/circos-schedule). A core set of evaluation questions are asked at the conclusion of each workshop using SurveyMonkey (https://www.surveymonkey.com/) (S3 Table). Recordings and links to relevant online documents are uploaded to YouTube (https://www.youtube.com/AustralianBioCommons).

## Training workshops

**Fig 2:**
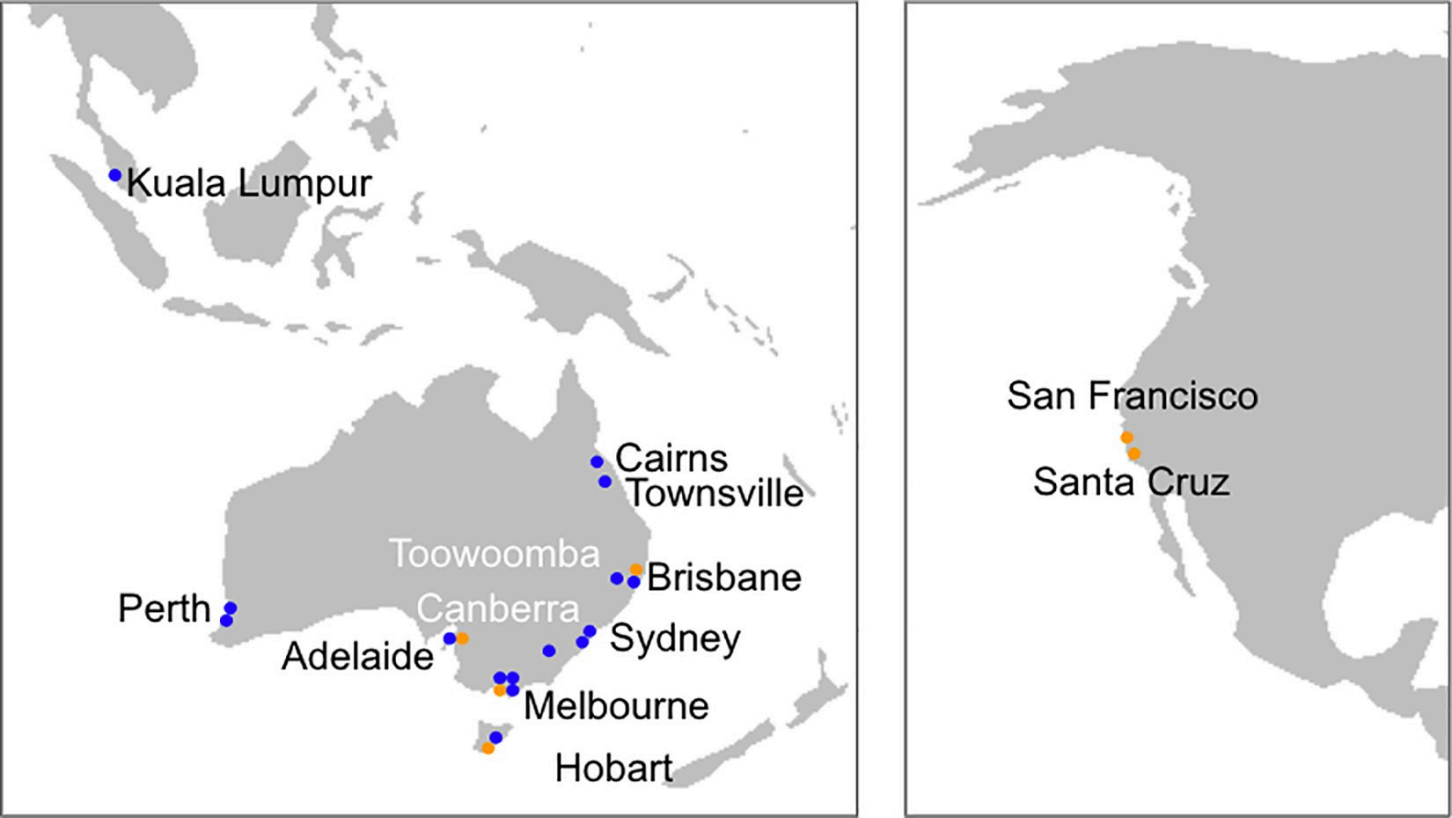
Locations of trainers and venues in hybrid bioinformatics training events. Locations of the trainers (orange dots) and venues with participants and facilitators (blue dots) are shown for the 9 hybrid bioinformatics training events held between 2017 and 2020. *Map source*: https://commons.wikimedia.org/wiki/File:BlankMap-World-noborders.png.

A variety of topics have been delivered as hybrid training events, engaging venues across Australia, Malaysia, and the USA. Between 2017 and 2020, over 800 participants attended live events on a range of platforms including Apollo [14], Galaxy Australia (https://usegalaxy.org.au/), the UCSC Genome Browser [15], and virtual SLURM (https://slurm.schedmd.com/) clusters deployed on Amazon Web Services cloud computing infrastructure (https://aws.amazon.com/). See Table 1 and Fig 2.

## Lessons learned

Participants were surveyed at the conclusion of each event (S3 Table), and facilitators and trainers provided feedback via group debriefs and individual conversations with the convenors. As experienced trainers and researchers themselves, facilitators were a valuable source of iterative improvements as the model was developed.

### Advantages

#### Access to training expertise

Hybrid training provides a low-barrier mechanism for sites across the country to access bioinformatics training expertise, enabling remote or small populations of researchers to interact directly with peers who can supplement local training programs. It allows expert trainers to easily reach new audiences, and the provision of the recordings and materials after the event allows for continued participant use. These freely available online resources may be suitable for reuse by trainers or self-guided use by the public.

#### Scaling and cost

Centralised coordination reduces the administrative burden of participation, and scaling to include more sites is simple once local facilitators have been identified. On average, each event had 90 participants. Trainers and facilitators have volunteered their time, and all events have been free of charge. Using this model, the cost to deliver the training was relatively low, even in circumstances when the trainer and facilitators were flown to a central location for a face-to-face training session prior to the public event.

#### Agility

The organisation of national training events is streamlined by coordinating with a large cohort of facilitator alumni. Experience in previous hybrid training events enables the quick instigation of new training activities as the opportunity arises.

#### Recognition for facilitators

This methodology fosters the professional development of trainers and facilitators who participate. It increases their visibility and can help to elevate their profile as local experts through their ability to provide new training opportunities. Hybrid training builds a community interested in bioinformatics training by creating new engagement between researchers and bioinformaticians.

### Challenges

Due to time zone differences across Australia of up to 4 hours, sessions are generally limited to 4 hours per day. While this ensures that participation is kept within business hours regardless of location, it does limit the potential learning outcomes. If the training topic requires more depth, multiple days are offered.

The hybrid training method also depends on the availability of reliable internet access. Australia’s stable research telecommunications network infrastructure (AARNet) interconnects all Australian research organisations nationally and globally across ultra high-speed (10 and 100 gigabit per second) links (https://www.aarnet.edu.au/network-and-services/the-network). Previously published methods have been described to optimise distance-based bioinformatics training in settings that lack stable internet access [11,12].

Multiple platforms and tools have been used to underpin training. For both users and administrators, the best choice is a publicly accessible hosted service that requires no specific setup, is resourced to support >100 concurrent users, and is available after the workshop (e.g., Galaxy Australia and UCSC Genome Browser). In cases where such a system is not available, cloud-based deployments can be utilised where all participants have access to identical setups, without the need for software installations on individuals’ devices.

Generating new training opportunities is most efficient with a stable and responsive group of facilitators who bring prior understanding of the processes required to implement training within their own venues. Many facilitators are researchers who need support to make connections with local research communities and institutional contacts who would benefit from the training offered. Early advertising of events in a range of communication channels is advantageous.

Large audiences can challenge trainers with both the volume and breadth of specialist questions. It is recommended that the trainer’s assistant is also a subject-matter expert. Deeper engagement of facilitators brings diverse expertise to the training materials and discussion board where technical questions are addressed in real time.

In 2020, due to the Coronavirus Disease 2019 (COVID-19) pandemic, assembling participants in classrooms has not been possible. Substituting virtual breakout rooms for physical classrooms has required a different ratio of facilitators to participants (1:5 versus 1:10) to ensure engagement remains high. Virtual breakout rooms that include both facilitators and participants from 1 organisation/site can still enable the building of local connections.

## Looking forward

The hybrid method efficiently delivers high-quality training to dispersed participants and will form an important part of the Australian BioCommons training program into the future. It is a valuable tool for sharing highly interactive events, providing access to expertise and reaching trainees without travel.

The Australian BioCommons training program is made possible by the participation of exceptional volunteer trainers and facilitators. Recognition of their contributions is always explicitly stated during live events, and further incentivisation will be required to sustain long-term engagement. The hybrid method is evolving through engagement with initiatives who understand the challenge of enduring engagement and the risk of volunteer fatigue, such as the Global Organisation for Bioinformatics Learning, Education and Training (https://mygoblet.org/), the International Society for Computational Biology (https://www.iscb.org/), and the Australian Bioinformatics and Computational Biology Society (https://www.abacbs.org/).

The hybrid training methodology is applicable to other disciplines, and there has been widespread interest from international and local colleagues to implement the hybrid training methodology into their own training activities (e.g., https://ardc.edu.au/project/humanities-arts-and-social-sciences/). The rapid development of other online training methods by organisations such as The Carpentries (https://carpentries.org/online-workshop-recommendations/) will no doubt offer many valuable insights for further development.

## Supporting information

S1 Checklist Hybrid training checklist and templates. (DOCX)

S1 Table Examples of bioinformatics training initiatives. (DOCX)

S2 Table Examples of current and previous Australian bioinformatics training initiatives and resources. (DOCX)

S3 Table Evaluation questions and responses. (DOCX)

## References

[1] Drysdale R, Cook CE, Petryszak R, Baillie-Gerritsen V, Barlow M, Gasteiger E, et al. The ELIXIR Core Data Resources: fundamental infrastructure for the life sciences. Bioinformatics. 2020 4 15;36(8):2636. 10.1093/bioinformatics/btz959 31950984 PMC7446027

[2] Sayers EW, Beck J, Bolton EE, Bourexis D, Brister JR, Canese K, et al. Database resources of the national center for biotechnology information. Nucleic Acids Res. 2021 1 8;49(D1):D10. 10.1093/nar/gkaa892 33095870 PMC7778943

[3] Batut B, Hiltemann S, Bagnacani A, Baker D, Bhardwaj V, Blank C, et al. Community-driven data analysis training for biology. Cell Syst. 2018 6 27;6(6):752–8. 10.1016/j.cels.2018.05.012 29953864 PMC6296361

[4] Barone L, Williams J, Micklos D. Unmet needs for analyzing biological big data: A survey of 704 NSF principal investigators. PLoS Comput Biol. 2017 10 19;13(10):e1005755. 10.1371/journal.pcbi.1005755 29049281 PMC5654259

[5] Stevens SL, Kuzak M, Martinez C, Moser A, Bleeker P, Galland M. Building a local community of practice in scientific programming for life scientists. PLoS Biol. 2018 11 28;16(11):e2005561. 10.1371/journal.pbio.2005561 30485260 PMC6287879

[6] Watson-Haigh NS, Shang CA, Haimel M, Kostadima M, Loos R, Deshpande N, et al. Next-generation sequencing: a challenge to meet the increasing demand for training workshops in Australia. Brief Bioinform. 2013 9 1;14(5):563–74. 10.1093/bib/bbt022 23543352 PMC3771231

[7] McGrath A, Champ K, Shang CA, van Dam E, Brooksbank C, Morgan SL. From trainees to trainers to instructors: Sustainably building a national capacity in bioinformatics training. PLoS Comput Biol. 2019 6 27;15(6):e1006923. 10.1371/journal.pcbi.1006923 31246949 PMC6597034

[8] Watson-Haigh NS, Revote J, Suchecki R, Tyagi S, Corley SM, Shang CA, et al. Towards an open, collaborative, reusable framework for sharing hands-on bioinformatics training workshops. Brief Bioinform. 2017 3 1;18(2):348–55. 10.1093/bib/bbw013 26984618 PMC5444239

[9] Revote J, Watson-Haigh NS, Quenette S, Bethwaite B, McGrath A, Shang CA. Development of a cloud-based Bioinformatics Training Platform. Brief Bioinform. 2017 5 1;18(3):537–44. 10.1093/bib/bbw032 27084333 PMC5429016

[10] Schneider MV, Griffin PC, Tyagi S, Flannery M, Dayalan S, Gladman S, et al. Establishing a distributed national research infrastructure providing bioinformatics support to life science researchers in Australia. Brief Bioinform. 2019 3;20(2):384–9. 10.1093/bib/bbx071 29106479 PMC6433737

[11] Mulder NJ, Adebiyi E, Alami R, Benkahla A, Brandful J, Doumbia S, et al. H3ABioNet, a sustainable pan-African bioinformatics network for human heredity and health in Africa. Genome Res. 2016 2 1;26(2):271–7. 10.1101/gr.196295.115 26627985 PMC4728379

[12] Gurwitz KT, Aron S, Panji S, Maslamoney S, Fernandes PL, Judge DP, et al. Designing a course model for distance-based online bioinformatics training in Africa: The H3ABioNet experience. PLoS Comput Biol. 2017 10 5;13(10):e1005715. 10.1371/journal.pcbi.1005715 28981516 PMC5628786

[13] Carvalho-Silva D, Garcia L, Morgan SL, Brooksbank C, Dunham I. Ten simple rules for delivering live distance training in bioinformatics across the globe using webinars. 10.1371/journal.pcbi.1006419 PMC6237289 30439935

[14] Dunn NA, Unni DR, Diesh C, Munoz-Torres M, Harris NL, Yao E, et al. Apollo: Democratizing genome annotation. PLoS Comput Biol. 2019 2 6;15(2):e1006790. 10.1371/journal.pcbi.1006790 30726205 PMC6380598

[15] Haeussler M, Zweig AS, Tyner C, Speir ML, Rosenbloom KR, Raney BJ, et al. The UCSC genome browser database: 2019 update. Nucleic Acids Res. 2019 1 8;47(D1):D853–8. 10.1093/nar/gky1095 30407534 PMC6323953

