# Supplementary material for: Application of a bioinformatics training delivery method for reaching dispersed and distant trainees
Source: PLoS Comput Biol. 2021 Mar 18;17(3):e1008715. doi: 10.1371/journal.pcbi.1008715 (PMC7971692; doi:10.1371/journal.pcbi.1008715)
Supplement: S1 Checklist — (DOCX) [file pcbi.1008715.s001.docx]

# S1 Checklist. Hybrid training checklist and templates

# Checklist for proposing a **training course**

## Required

- Identify a **lead trainer** to deliver the training and confirm they are available for the proposed **training course**
- A written outline of the **training course** that includes:
  - intended audience (discipline and level of expertise)
  - general content that will be covered
  - learning objectives and prerequisites
  - a training schedule (allowing for plenty of time for local **facilitators** to set-up, demonstration from **lead trainer**, and hands-on exercises with time for local **facilitators** to re-group with their node).
- Willingness to have the **training course** recorded and made available via the organisation's YouTube channel and any other appropriate channels.
- A commitment to conduct a **train the facilitator** session several weeks prior to the actual **training course** with **facilitators** at each participating **training node**
- At least three proposed dates/times that the **training course** could be offered
- At least three proposed dates/times that the **train the facilitator** could be offered (2-3 weeks prior to the actual **training course**)

## Desirable

- Introductory presentation material (slides etc)
- Set of exercises
- A suitable on-line training environment with up to 150 user accounts (preferred) or an easily installable tool
- Due to time zone differences across Australia, short **training courses** (half day i.e. max 4 hours with breaks) are preferred.

Time frame for submission of the proposal: at least 2 months prior to the intended **training course**.

# General preparation for a **training event**

## Preparation (D - 12 weeks)

- **Convenor** receives an email when a web form for **training course** proposal is submitted
- **Convenor** creates a Google Drive folder for the event
- **Convenor** deposits a copy of the proposal in the Google Drive folder
- **Convenor** reviews the content of the proposal and provides feedback to the proposer asking for clarifications as required

## General preparation (D - 10/12 weeks)

- **Convenor** checks organisation's training and other meeting calendars for clashes with proposed dates for the **training course**
- **Convenor** checks their availability to host on the proposed dates/times
- **Convenor** prepares Doodle poll with the three proposed times for **train the facilitator**
- **Convenor** prepares Doodle poll with the three proposed times for **training course**
- **Convenor** invites **training nodes** to complete the 2 Doodle polls to determine the final date and times of the **train the facilitator** and **training course**
- **Training nodes** complete Doodle polls
- Once dates are finalised, **convenor** sends a copy of the RSVP web-form that is mandatory for participating **training nodes** (to get names of **facilitators** and details of the venue)
- **Training nodes** complete RSVP form including names/emails of **facilitators** and venue details
- Once dates are finalised, **convenor** prepares placeholder calendar invites using the organisation's shared Google Calendar for **training the facilitator** and the **training course**

## General preparation (D - 4/10 weeks)

- **Convenor** sends an email to the lead **trainer** thanking them for agreeing to participate, with clear details of the **train the facilitator** and the **training course**, and includes the "permission to publish" statement.
- **Convenor** sends a draft event run-sheet and links to example material to the **lead trainer** as needed.
- **Lead trainer** sends Introductory presentation material (slides etc) to the **convenor**
- **Lead trainer** sends copy of exercises to the **convenor**
- **Lead trainer** reconfirms that a suitable on-line training environment with up to 150 user accounts (preferred, and assuming 15 attendees at 10 nodes) OR an easily installable and reliable tool is available
- **Lead trainer** sends their credentials to the **convenor** to test log-in into any proposed training environment/platform
- **Lead trainer** and **facilitators** confirm willingness to have the **training course** recorded and made available via the organisation's YouTube channel and any other appropriate channels
- **Convenor** puts all items received from **lead trainer** in the Google Drive folder
- **Convenor** confirms receipt of "permission to publish" statement from **lead trainer** and saves copy in the Google Drive folder
- **Convenor** schedules a Zoom teleconference for the **training course**
- **Convenor** updates the calendar invite using the organisation's shared Google Calendar and includes links to any prerequisite material and the URL for the Zoom teleconference
- **Convenor** sends calendar invite to **lead trainer**
- **Convenor** sends calendar invite to all **facilitators**
- **Convenor** sends calendar invite to the organisation
- **Convenor** prepares draft Eventbrite registration page and sends to **lead trainer** to review **training course** description and proposed registration survey questions
- **Lead trainer** confirms that draft Eventbrite registration page is correct
- **Convenor** sends draft Eventbrite registration page to **facilitators** to review local details such location, local time etc.
- **Facilitators** confirm that draft Eventbrite registration page is correct for their **training node**
- **Convenor** sends link to each final Eventbrite registration page to each participating **training node**
- **Convenor** prepares advertising material (for email) that includes:
  - TITLE
  - SYNOPSIS
  - TOPICS TO BE COVERED
  - WHEN
  - WHERE (which includes links to all Eventbrite registration pages)
  - WHAT TO BRING
- **Training nodes** advertise **training course** locally
- **Convenor** advertises **training course** via Twitter
- **Convenor** advertises **training course** via the organisation's website
- **Convenor** advertises **training course** via the organisation's newsletter

# Organising the **train the facilitator** event

## Preparation (D - 4/6 weeks)

- **Convenor** sends email to **lead trainer** thanking them, with clear details of the **train the facilitator** event
- **Lead trainer** sends any prerequisite material for **train the facilitator** to the **convenor**
- **Lead trainer** reconfirms that a suitable on-line training environment with enough user accounts for all **facilitators** is (or will be) available
- **Convenor** schedules a Zoom teleconference for the **train the facilitator** event
- **Convenor** prepares a calendar invite using the organisation's shared Google Calendar with links to any prerequisite materials and the URL for the Zoom teleconference
- **Convenor** sends calendar invite to **lead trainer**
- **Convenor** sends calendar invite to all **facilitators**
- **Convenor** sends calendar invite to the organisation

## Preparation (train the facilitator - 1 week)

- **Convenor** provides names of facilitators to the **lead trainer**

## On the day

- **Convenor** hosts Zoom teleconference, introduces the **lead trainer** and **facilitators** in participating **training nodes** and the logistics for the session.
- **Convenor** records Zoom teleconference
- **Lead trainer** trains the **facilitators**
- **Lead trainer** ensures the schedule is discussed/shared with **facilitators**

## After the day

- **Convenor** edits recording of Zoom teleconference and uploads it to YouTube (privately)
- **Convenor** distributes link to YouTube recording to **lead trainer** and **facilitators** (especially any **facilitators** who could not attend)
- **Lead trainer** responds to any questions the **facilitators** may have

# Organising the **training course**

## Preparation (D - 1 week)

- **Convenor** sends reminder to registrants reminding them of the **training course** and encouraging them to cancel registration if no longer able to attend
- **Convenor** informs **facilitators** of latest registration numbers and Eventbrite registration survey responses of registrants, including sign-in sheet and timing schedule
- **Convenor** reminds **facilitators** they need to get pink/red and green post-it notes for flagging purposes
- **Convenor** informs **lead trainer** of latest registration numbers and Eventbrite registration survey responses of registrants
- **Lead trainer** ensures sufficient training accounts are resourced
- **Training nodes** further advertise **training course** as required
- **Training nodes** ensure training environment and all material can be accessible by attendees using the method the attendees will be using on the day (e.g. on a BYO laptop in the actual room over local wi-fi)
- **Convenor** drafts follow-up post-**training course** email message to attendees
- **Convenor** set-ups post-**training course** SurveyMonkey questionnaire
- **Convenor** prepares **convenor** run-sheet
- **Convenor** prepares **facilitators** run-sheet
- **Convenor** prepares introductory slides and deposits them in Google Drive

## Preparation (D - 1 day)

- **Convenor** closes registration 24 hours prior to the start of **training course**
- **Lead trainer** assigns training accounts to attendees as required
- **Lead trainer** sends a copy of slides to the organisation
- **Convenor** sends a reminder to registrants that the **training course** is on the following day
- **Convenor** informs **lead trainer** and **facilitators** of final information on attendees (numbers, demographics etc.)
- **Convenor** sends check-in list to **facilitators**
- **Convenor** ensures training accounts: user assignment information are distributed to **Lead trainer** and **facilitators** as required
- **Convenor** sets up a discussion board for the **training course** and adds standard text: what it’s for, etiquette of its use, adhering to the organisation's Code of Conduct
- **Training nodes** print material as required

# Running the **training course**

## On the day (D - 1 hour)

- **Convenor** start Zoom teleconference
- **Training nodes** join Zoom teleconference
- **Training nodes** conduct video and sound checks

## On the day (D - 15/30 minutes)

- **Facilitators** welcome attendees to venue
- **Facilitators** get attendees settled in the room and logged onto wi-fi and any system they will be using
- **Convenor** sends a link to the discussion board to **lead trainer**, **facilitators** and attendees via email
- **Facilitators** get attendees onto the discussion board
- **Facilitators** distribute post-it notes to attendees
- **Facilitators** check-in **training course** attendees using check-in list

## During the training course

- **Lead trainer** delivers training according to the agreed schedule and allowing plenty of time for local **facilitators** to set-up, demonstration from **lead trainer**, and hands-on exercises with enough time for **facilitators** to re-group with their local attendees.
- **Facilitators** facilitate at their **training node**, encourage the use of post-it notes, field any local attendee questions and encourage attendees to write questions into the discussion board
- **Facilitators** keep their **training node** muted on Zoom
- **Convenor** manages Zoom logistics
- **Convenor** mutes/unmutes **training nodes**
- **Convenor** monitors the discussion board and manages communication between **lead trainer** and each **training node**
- Attendees can flag issues using Post-it notes which **facilitators** can help with
- Attendees can raise issues using the discussion board
- **Lead trainer** addresses issues raised on the discussion board

## Prior to the end (30 minutes)

- **Convenor** flags the time with the **lead trainer**

## Prior to the end (10 minutes)

- **Convenor** flags the time with the **lead trainer**
- **Lead trainer** wraps things up
- **Lead trainer** hands over to the **convenor**
- **Convenor** wraps up and thanks **lead trainer**, **facilitators** and attendees
- **Convenor** refers to the survey on the discussion board and encourages attendees to complete it (allowing 5 minutes)
- **Convenor** verbally lets attendees know of follow-up materials to come

## At the end of the training course

- **Facilitators** finish things at their **training nodes**
- **Convenor** stops recording Zoom and downloads the recording to the local hard drive (MP4 format)
- **Facilitators** send numbers/check-in stats to the **convenor**

# After the **training course**

## Recording

- **Convenor** puts a copy of the recording into event folder on Google Drive
- **Convenor** edits the video recording of the event (QuickTime)
- **Convenor** uploads edited video to the organisation's YouTube channel
- **Convenor** sends link to the **lead trainer** for checking (with 2-day turnaround to decline to publish)
- **Convenor** checks permissions to publish statements are all in the relevant Google Drive folder
- **Convenor** checks descriptions of the video on YouTube
- After the 2-day turnaround, **convenor** changes YouTube video permissions from Unlisted to Public
- **Convenor** puts video links in slides descriptions
- **Convenor** puts video links on the organisation's website

## Slides

- **Convenor** gets slides from **lead trainer**
- **Convenor** adds slides to Slideshare
- **Convenor** sends Slideshare links to the organisation's communications team
- **Convenor** puts slides link in the YouTube description
- **Convenor** puts YouTube link in the slides description
- **Convenor** puts **training course** description in the slides description
- **Convenor** puts slide links on the organisation's website

## Surveys and reports

- **Convenor** downloads Survey Monkey reports to Google Drive folder
- **Convenor** add ideas for future **training courses** to backlog of ideas
- **Convenor** forwards any question/comments on to **lead trainer** as appropriate

## Final follow-ups

- **Convenor** drafts an email to attendees using template
- **Convenor** sends out the email to attendees - bcc'ing all attendees
- **Convenor** sends out a social media message with links to recordings etc.
- **Convenor** send links to recordings to be included in the organisation's next newsletter
- **Convenor** emails thank-you notes to **lead trainer** and **facilitators** and cc's managers as appropriate
- **Convenor** reflects at the organisation's team meeting
- **Convenor** checks attendee evaluations for comments and passes them on as appropriate
- **Convenor** closes off folder on Google Drive - including image of permission to publish statements, presentations, final registrations spreadsheet, delete all versions of registration lists, draft documents etc.

Templates

# Registration info for attendees

- Training node
- Order Date
- First Name
- Surname
- Email
- Gender
- Country
- Nationality
- Are you part of the network?
- Are you a: (researcher / professional / ...)
- What do you call yourself?
- Which topic/s do you work on?
- What are the top three digital resources/tools you use?
- What do you expect to gain from this workshop?
- How did you hear about this workshop?
- What was your source?
- Job Title
- Company

# Workshop evaluation by attendees

1. What is your job role?
2. Where did you participate in the workshop?
3. This was a useful workshop that enhanced my knowledge and skills (on a scale of 1-5)
4. The content reflected the learning objectives of the workshop (on a scale of 1-5)
5. The learning from this program increased my level of skills (on a scale of 1-5)
6. The program was delivered in a sequence that was meaningful (on a scale of 1-5)
7. The exercises and activities enhanced my learning (on a scale of 1-5)
8. The course duration was adequate to cover the content (on a scale of 1-5. If you choose 1-3, how long should it be?)
9. The pace of the course was adequate for my learning (on a scale of 1-5. If you choose 1-3, should it be slower or faster?)
10. There was a broad enough range of activities to encourage participation (on a scale of 1-5)
11. The lead trainer was organised and confident in the program content (on a scale of 1-5)
12. The lead trainer was responsive to individual questions and needs (on a scale of 1-5)
13. My local facilitator was helpful and confident in the program content (on a scale of 1-5)
14. The live Discussion Board was useful during the workshop (on a scale of 1-5)
15. Would you like to add any other comments, or do you have suggestions for improvements in future workshops?

# Workshop opening slides

## Slide 1

Welcome

the training will commence at

<TIME in different time zones>

While you are waiting - bookmark today’s schedule…

<URL>

## Slide 2

<Title of the workshop>

## Slide 3

<Background on the organisation and its distributed nature>

## Slide 4

Today's training:

<Topics covered and location of training nodes>

## Slide 5

Lead trainer

<NAME>

## Slide 6

Facilitators

<NAMES>

## Slide 7

Getting involved

All sites will be in listen-only mode while the speakers are talking

Ask your Local facilitator for help / Green = All Good / Pink = Please Help!

Ask the lead trainer questions using the Discussion Board: <URL>

The session will be recorded and selected parts broadcast via the organisation's YouTube channel

## Slide 8

Next Training Event

<URL to training website>

<Schedule>

<URL to YouTube channel>

## Slide 9

Feedback

<Screenshot of where to find URL of evaluation survey>

## Slide 10

Workshop sponsors

# Event overview template

## TITLE

## SYNOPSIS

A couple of descriptive sentences.

FORMAT

The workshop has been organised by XXX and will be led by YYY from ZZZ who is AAA.

XXX will be joining us direct from YYY, and the workshop will occur simultaneously in a number of venues across Australia where we will have locally trained facilitators on hand to help and facilitate the workshop locally.

## INTENDED AUDIENCE

Who:

Prerequisite knowledge:

## LEARNING OUTCOMES

By the end of this training, participants will know / be able to:

## TOPICS TO BE COVERED

**Xxx Xxx**

- Yyy yyy
- Zzz Zzz

**Xxx Xxx**

- Yyy yyy
- Zzz Zzz

## WHEN

- Day, date, time (if known)

## EVENT SCHEDULE

| Time | Activity |  |
| --- | --- | --- |
| Eg. 1.00pm | Welcome and speaker intro | Convenor |
| Eg. 1.10pm | Intro workshop topic and start talk component | XXX |
| Eg. 2.00pm | Intro hands-on exercises 1-3, allow 10 mins for participants to complete (speaker offline) | Participants |
| Eg. 2.10pm | Speaker returns and requests questions from facilitators, runs through answers | Speaker XXX and facilitators |
| Eg. 2.30pm | Speaker continues talking | XXX |
| Eg. 3.00pm | Intro exercises 4-5 and allow 10 mins to complete | Participants |
| etc... |  |  |
| Eg. 4:50pm | Wrap up | Convenor |

## PARTICIPANT REQUIREMENTS

Eg. Please bring a laptop with an up to date web browser (Chrome or Firefox), and ensure you are able to log in to [eduroam](https://www.eduroam.edu.au).

# Training Event Proposal

This webform is used to propose a hybrid training course or workshop (i.e. with a remote presenter and local training venues at participating Nodes) via the organisation’s network.

We'll use the information you provide here to decide on a date/time for the training, invite other nodes to participate, discern whether your material and training environment are ready for delivery or require further work, and prepare general advertising and registration information for the event.

You will be sent an email after submitting the response and will be able to update any of your responses using a link provided in that email.

We'll be in touch shortly after submission to discuss your event further.

Many thanks

The organisation

*Required

Email address *

Your name *

Your Node *

Training event content

Please provide information about the event (in language that would be suitable for advertising purposes).

Title *

Description/synopsis *

Topics to be covered *

Level *

Novice

Intermediate

Expert

Pre-requisites for attendance *

e.g. familiarity with the UNIX command line, or an understanding of basic statistical methods is required.

Materials and Resources

For a training event, the following materials/resources are required: A written outline of the course, a training schedule, introductory presentation material, a set of exercises, data files and a suitable training environment.

Course outline *

Please provide a URL to a written course outline

Training schedule/timetable *

Please provide a URL to a schedule/timetable for the proposed event. (Notes: Due to time-zone differences across Australia, max. 4hrs content is preferred; allow plenty of time for (a) local facilitators at each node to set-up, (b) for demonstrations from your lead trainer, and (c) hands-on exercises with time for local facilitators to re-group with their node.)

Introductory Slides

Please provide a URL to your introductory slides

Exercises

Please provide a URL to the exercises

Data files

Please provide a URL to download links for any data files required for the exercises

Training environment *

A suitable on-line training environment (preferred) with up to 150 user accounts or an easily installable tool is required. Please provide a URL to whichever is relevant.

Training environment log-in details

If a log-in is required for the environment, please provide us with a username and password so we can test the accessibility of the system

Event Logistics

Training Leader name *

The name of the person who will lead the training across the network via zoom

Training Leader email address *

Requirements for organisers *

I will provide a familiarisation session for local Node facilitators several weeks prior to the actual event.

I understand the training materials will be made available for future re-use (licensed under a suitable Creative Commons License e.g. http://creativecommons.org.au/learn/licences/).

Both I and the training leader understand the familiarisation and training events will be recorded and made available via the organisation’s YouTube channel and/or any other appropriate channels.

Proposed dates/times: Training event

The following will be used to invite participation from other Nodes.

Please take into consideration time-zone differences across Australia when proposing start and end times.

Please propose dates at least 2 months away to ensure we have sufficient time to organise and advertise the event.

Time zone used in this form *

Please specify the time zone you are using to denote the below any times below

AEST Australian Eastern Standard Time

AEDT Australian Eastern Daylight Time

ACST Australian Central Standard Time

ACDT Australian Central Daylight Time

AWST Australian Western Standard Time

Proposed Date # 1 *

Date

Start and End Times *

Proposed Date # 2 *

Date

Start and End Times *

Proposed Date # 3 *

Date

Start and End Times *

Proposed dates/times: Local facilitator familiarisation session

This session will be used to familiarise trainers at each node in the course content, tool functionalities etc 2-3 weeks prior to the actual training event.

Time zone used in this form *

Please specify the time zone you are using to denote the below any times below

AEST Australian Eastern Standard Time

AEDT Australian Eastern Daylight Time

ACST Australian Central Standard Time

ACDT Australian Central Daylight Time

AWST Australian Western Standard Time

Proposed Date # 1 *

Date

Start and End Times *

Proposed Date # 2 *

Date

Start and End Times *

Proposed Date # 3 *

Date

Start and End Times *

Any other comments/questions

Please use this to add any other comments/requirement/questions etc

Comments etc

A copy of your responses will be emailed to the address that you provided.

# Hybrid Training Event RSVP

This webform is used by an Node to RSVP to an invitation to participate in a hybrid training event.

It contains a checklist for what will be required at your node to participate.

We'll use some of the information (e.g. training room location) to prepare Eventbrite invites for you and the names and email addresses of your facilitators for further correspondence.

Many thanks

The organisation

*Required

Email address *

Your name *

Your Node *

Local facilitators

An expert lead trainer will be delivering the training over Zoom.

Local facilitators will be required at your node to book appropriate rooms, advertise locally, be responsible for logistics at your node on the day and field any basic questions from attendees at your node during the event.

We recommend having at least 1 facilitator for every 10 attendees.

Facilitators will be required to attend a 'train the facilitator' session which will be held at least 2 weeks prior to the event.

Names of your facilitators *

Email addresses of your facilitators *

Training venue

You will need a suitable venue for the hybrid training which is held using zoom.

Features of the venue are:

- A capacity of 10 x number of local facilitators

- Either desktop machines with internet access and log-in options for all attendees, OR Eduroam available if attendees will bring their own lap-top

- A sufficiently large, and high resolution screen for the zoom presentations

- Capacity to join in a zoom videoconference (one for the room (for video and to see the lead trainer's presentation and desktop) and one for the facilitator (for chat directly with the lead trainer if required)

- And, a camera facing the audience to generate a sense of community across the country during the event

Venue address *

This address will appear in Eventbrite invitations that the organisation will make on your behalf, so please be as complete as possible

Venue capacity *

This will be used to set a registration cap for your venue in the Eventbrite invitations we will make on your behalf

A copy of your responses will be emailed to the address that you provided.

# Post training course email to attendees

*Subject:* training course: <Title>. Link to Recording and Slides

Thank you for registering for last week's training course: <Title>.

A recording of the presentation is available here <YouTube Link>

<Presenter Name>’s Powerpoint is here <slideshare link>

Links mentioned in the webinar:

<Add links to resources mentioned here>

Keep in touch:

To find out more about upcoming events, resources, news and more:

Subscribe to the organisation’s quarterly newsletter <link>

Join the conversation on Twitter: <handle>

We look forward to 'seeing’ you again at the next training course:

Title: <title>

Date/time: <date time, timezone >

Register: <registration link>

Speakers will reflect on <topic>

• <Speaker name, Speaker affiliation>
