# Supplementary material for: Application of a bioinformatics training delivery method for reaching dispersed and distant trainees
Source: PLoS Comput Biol. 2021 Mar 18;17(3):e1008715. doi: 10.1371/journal.pcbi.1008715 (PMC7971692; doi:10.1371/journal.pcbi.1008715)
Supplement: S1 Table — (DOCX) [file pcbi.1008715.s002.docx]

**S1 Table. Examples of bioinformatics training initiatives**

The scale of each initiative is shown (i.e. global/continental/national or institutional), as well as the features/ generated outputs.

| **Initiative/Resource** | **URL** | **Scale** | Face to face training workshops | On-line (self- paced) training | Community of trainers | Repository of training materials | Training events portal | Train the trainer program |
| --- | --- | --- | --- | --- | --- | --- | --- | --- |
| GOBLET - Global Organisation for Bioinformatics Learning, Education & Training | <https://www.mygoblet.org/> | Global | - | - | ✓ | ✓ | ✓ | - |
| The Carpentries | <https://carpentries.org/> | Global | ✓ | ✓ | ✓ | ✓ | ✓ | ✓ |
| Galaxy Training | <https://galaxyproject.github.io/training-material/> | Global | - | ✓ | ✓ | ✓ | - | - |
| LifeSciTrainingSlack | <https://jasonjwilliamsny.github.io/LifeSciTrainingSlack/> | Global | - | - | ✓ | - | - | - |
| GTPB (Gulbenkian Training Program in Bioinformatics) | <http://gtpb.igc.gulbenkian.pt/bicourses/index.html> | Global | ✓ | - | - | ✓ | ✓ | - |
| ELIXIR Training platform | <https://elixir-europe.org/platforms/training> | Continental | ✓ | ✓ | ✓ | ✓ | ✓ | ✓ |
| CABANA (Capacity Building for Bioinformatics in Latin America) | <https://www.cabana.online> | Continental | ✓ | ✓ | ✓ | - | ✓ | ✓ |
| H3ABioNet (Pan African Bioinformatics Network for H3 Africa) Training | <https://www.h3abionet.org/training> | Continental | ✓ | ✓ | ✓ | - | ✓ | - |
| European Bioinformatics Institute Training | <https://www.ebi.ac.uk/training> | Continental+ | ✓ | ✓ | - | ✓ | ✓ | ✓ |
| Bioinformatics.ca | <https://bioinformatics.ca/> | National | ✓ | - | ✓ | ✓ | ✓ | - |
| SIB (Swiss Institute of Bioinformatics) Training | <https://www.sib.swiss/training/upcoming-training-courses> | National | ✓ | ✓ | ✓ | - | ✓ | - |
| de.NBI (German Network for Bioinformatics Infrastructure) Training | <https://www.denbi.de/training> | National | ✓ | ✓ | ✓ | - | ✓ | - |
| DTL (Dutch Techcentre for Lifesciences) Training | <https://www.dtls.nl/courses/> | National | ✓ | - | - | - | ✓ | - |
| Genomics Aotearoa Training | <https://github.com/GenomicsAotearoa> | National | ✓ | - | ✓ | ✓ | ✓ | - |
| BD2K ERuDIte | <https://bigdatau.ini.usc.edu/> | National | - | - | - | ✓ | - | - |
| University of Cambridge Bioinformatics Training | <https://bioinfotraining.bio.cam.ac.uk/> | Institutional | ✓ | - | - | - | ✓ | - |
| Cyverse Learning | <https://learning.cyverse.org/en/latest/> | Global | ✓ | ✓ | - | ✓ | ✓ | - |
