# Supplementary material for: Application of a bioinformatics training delivery method for reaching dispersed and distant trainees
Source: PLoS Comput Biol. 2021 Mar 18;17(3):e1008715. doi: 10.1371/journal.pcbi.1008715 (PMC7971692; doi:10.1371/journal.pcbi.1008715)
Supplement: S2 Table — (DOCX) [file pcbi.1008715.s003.docx]

**S2 Table. Examples of current and previous Australian bioinformatics training initiatives and resources**

Selected examples range across (a) bioinformatics training programs that are intended for national, state or institutional audiences, (b) residential bioinformatics schools, (c) reusable frameworks that have been developed to deliver hands-on bioinformatics training, and (d) Train-the-trainer programs.

| **Initiative/Resource Type** | **Examples** | **Reference/URL** |
| --- | --- | --- |
| Nationally coordinated bioinformatics training | Australian Bioinformatics  Training Network Initiative (jointly coordinated by Bioplatforms Australia and the Commonwealth Scientific and Industrial Research Organisation (CSIRO) Bioinformatics Core. | <https://www.bioplatforms.com/bioinformatics-training/> |
| Bioinformatics training offered by State-based organisations | Queensland Facility for Advanced Bioinformatics (QFAB) Training | <https://qfab.org/training> |
| Bioinformatics training offered by Institutions | University of Sydney | <https://sydney.edu.au/research/facilities/sydney-informatics-hub/workshops-and-training.html#bioinformatics> |
|  | University of Melbourne | <https://www.melbournebioinformatics.org.au/training-and-events/> |
|  | Monash University | <https://www.monash.edu/researchinfrastructure/bioinformatics/training> |
|  | University of Adelaide | <https://www.adelaide.edu.au/bioinformatics-hub/training> |
|  | University of Western Australia | <http://www.appliedbioinformatics.com.au/index.php/Main_Page> |
| Introductory-level Residential Bioinformatics Schools | UQ (University of Queensland) Winter School in Bioinformatics | <http://bioinformatics.org.au/ws/> |
|  | AMSI (Australian Mathematical Sciences Institute) BioInfoSummer | <https://bis.amsi.org.au/> |
| Reusable frameworks for sharing and delivering hands-on bioinformatics training workshops | Bioinformatics Training Platform (BTP) | [8,9] |
| Train-the-trainer programs aligned with international exemplars | Bioplatforms Australia / CSIRO / EMBL-EBI Train-the-Trainer (TtT) programme | [7] |
