# Supplementary material for: Application of a bioinformatics training delivery method for reaching dispersed and distant trainees
Source: PLoS Comput Biol. 2021 Mar 18;17(3):e1008715. doi: 10.1371/journal.pcbi.1008715 (PMC7971692; doi:10.1371/journal.pcbi.1008715)

**S3 Table. Evaluation questions and responses**

Participant survey questions and statements

| **Question** | | **Response Option** |
| --- | --- | --- |
| Q1 | What is your job role? | 1. Group Leader  2. Post doctoral researcher  3. Postgraduate student  4. Undergraduate student  5. Service provider  6. Other |
| Q2 | Where did you participate in the workshop? | Choose from a list of participating sites |
| Q3 | This was a useful workshop that enhanced my knowledge and skills. | 1. No, I strongly disagree  2. No, I disagree  3. I neither disagree or agree  4. Yes, I agree  5. Yes, I strongly agree |
| Q4 | The content reflected the learning objectives of the workshop. | 1. No, I strongly disagree  2. No, I disagree  3. I neither disagree or agree  4. Yes, I agree  5. Yes, I strongly agree |
| Q5 | The learning from this program increased my level of skills. | 1. No, I strongly disagree  2. No, I disagree  3. I neither disagree or agree  4. Yes, I agree  5. Yes, I strongly agree |
| Q6 | The program was delivered in a sequence that was meaningful. | 1. No, I strongly disagree  2. No, I disagree  3. I neither disagree or agree  4. Yes, I agree  5. Yes, I strongly agree |
| Q7 | The exercises and activities enhanced my learning. | 1. No, I strongly disagree  2. No, I disagree  3. I neither disagree or agree  4. Yes, I agree  5. Yes, I strongly agree |
| Q8 | The course duration was adequate to cover the content. (If you  choose 1-3, how long should it be?) | 1. No, I strongly disagree  2. No, I disagree  3. I neither disagree or agree  4. Yes, I agree  5. Yes, I strongly agree |
| Q9 | The pace of the course was adequate for my learning. (If you choose  1-3, should it be slower or faster?) | 1. No, I strongly disagree  2. No, I disagree  3. I neither disagree or agree  4. Yes, I agree  5. Yes, I strongly agree |
| Q10 | There was a broad enough range of activities to encourage  participation. | 1. No, I strongly disagree  2. No, I disagree  3. I neither disagree or agree  4. Yes, I agree  5. Yes, I strongly agree |
| Q11 | The lead trainer was organised and confident in the program  content. | 1. No, I strongly disagree  2. No, I disagree  3. I neither disagree or agree  4. Yes, I agree  5. Yes, I strongly agree |
| Q12 | The lead trainer was responsive to individual questions and needs. | 1. No, I strongly disagree  2. No, I disagree  3. I neither disagree or agree  4. Yes, I agree  5. Yes, I strongly agree |
| Q13 | My local facilitator/s was/were helpful and confident in the program  content. | 1. No, I strongly disagree  2. No, I disagree  3. I neither disagree or agree  4. Yes, I agree  5. Yes, I strongly agree |
| Q14 | The live Discussion Board was useful during the workshop. | NA. I didn't look at the Discussion Board  1. No, I strongly disagree  2. No, I disagree  3. I neither disagree or agree  4. Yes, I agree  5. Yes, I strongly agree |
| Q15 | Would you like to add any other comments, or do you have  suggestions for improvements in future workshops? | Free text answer |

**Participant responses**

Aggregate participant responses across the nine hybrid training events described in this paper (number of responses = 365)


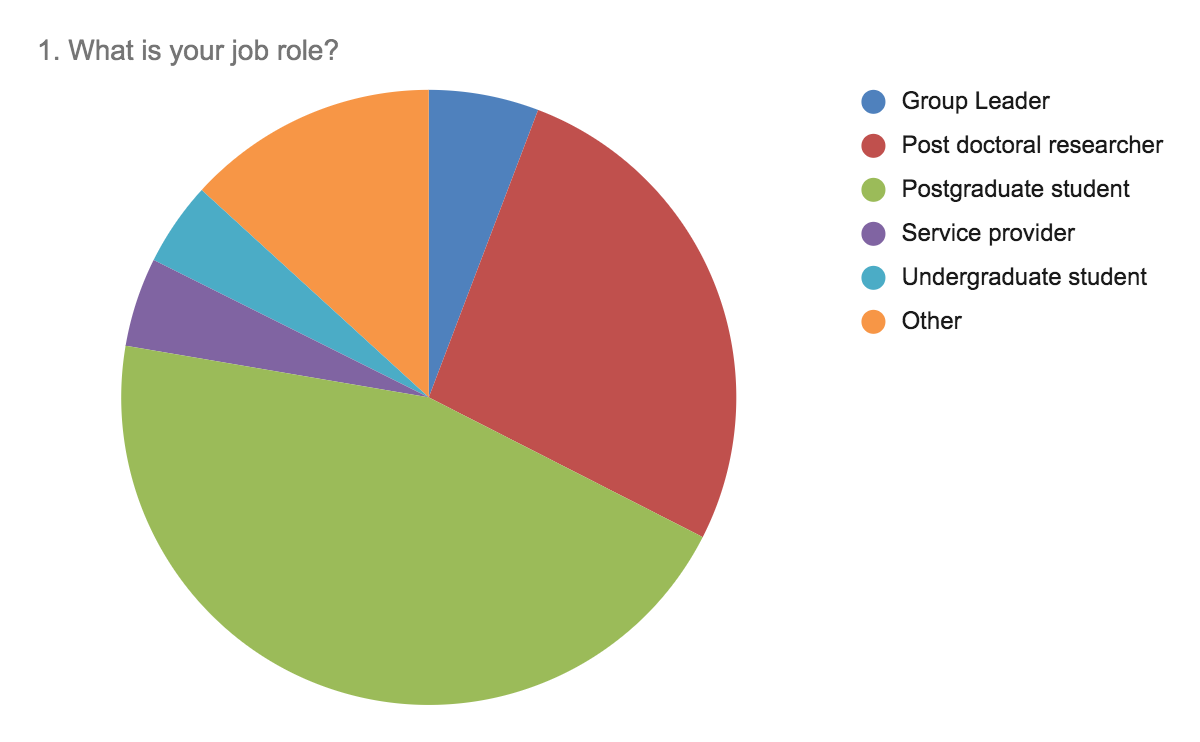


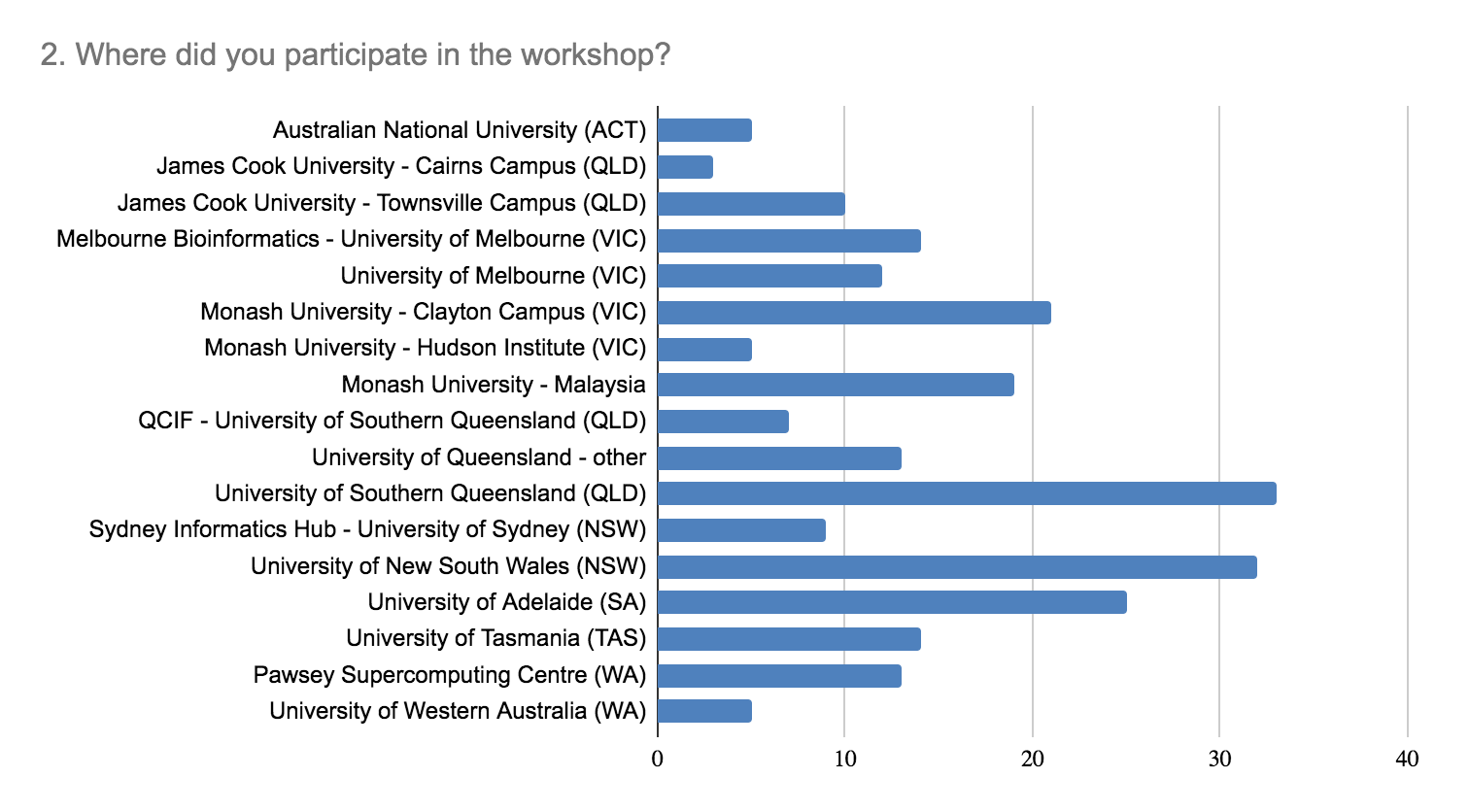


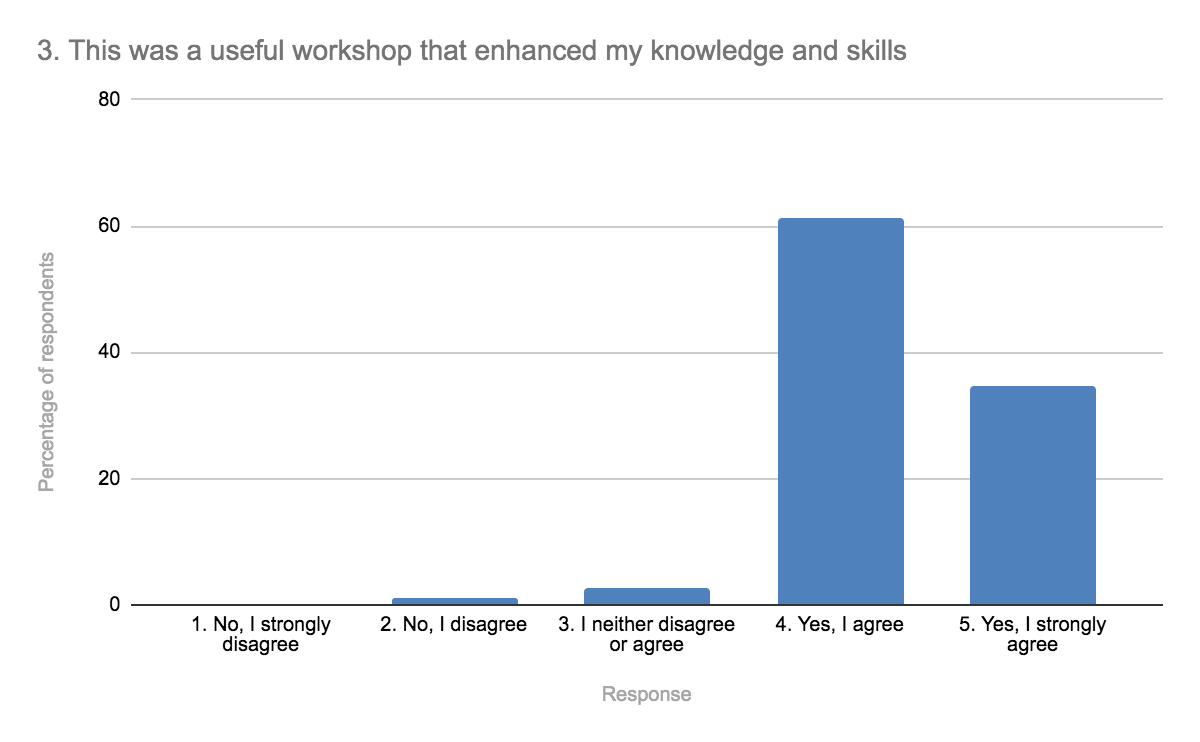


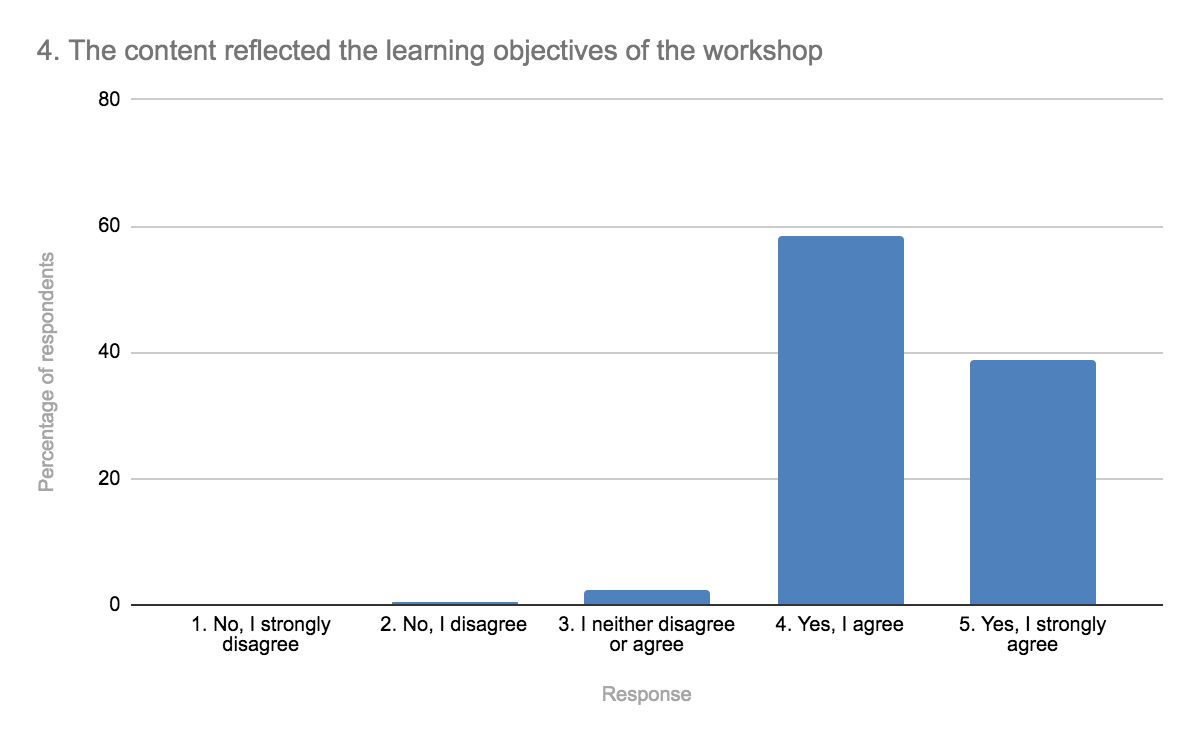


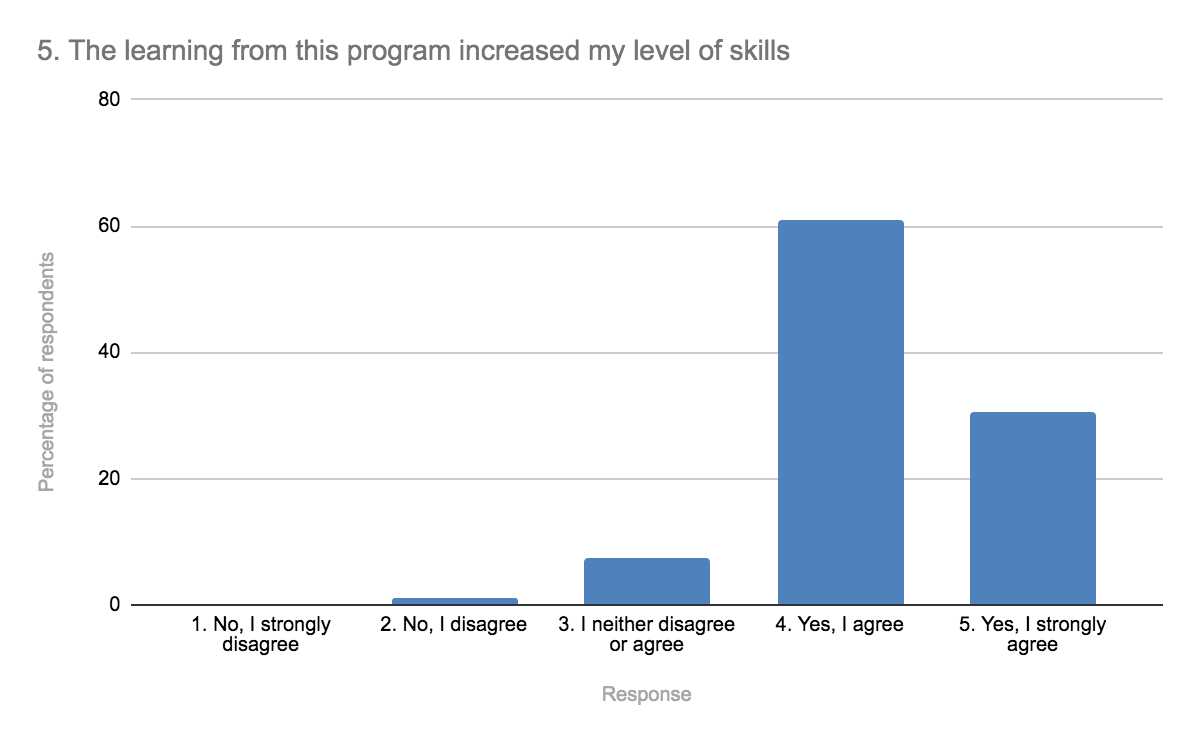


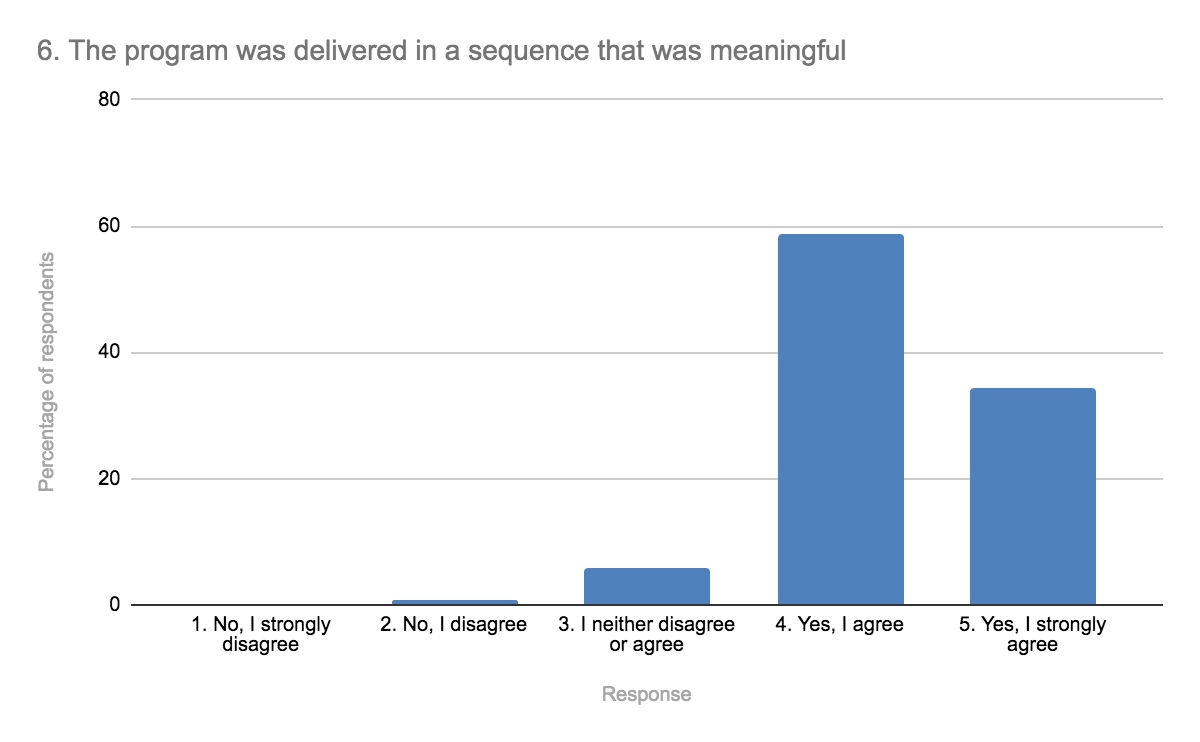


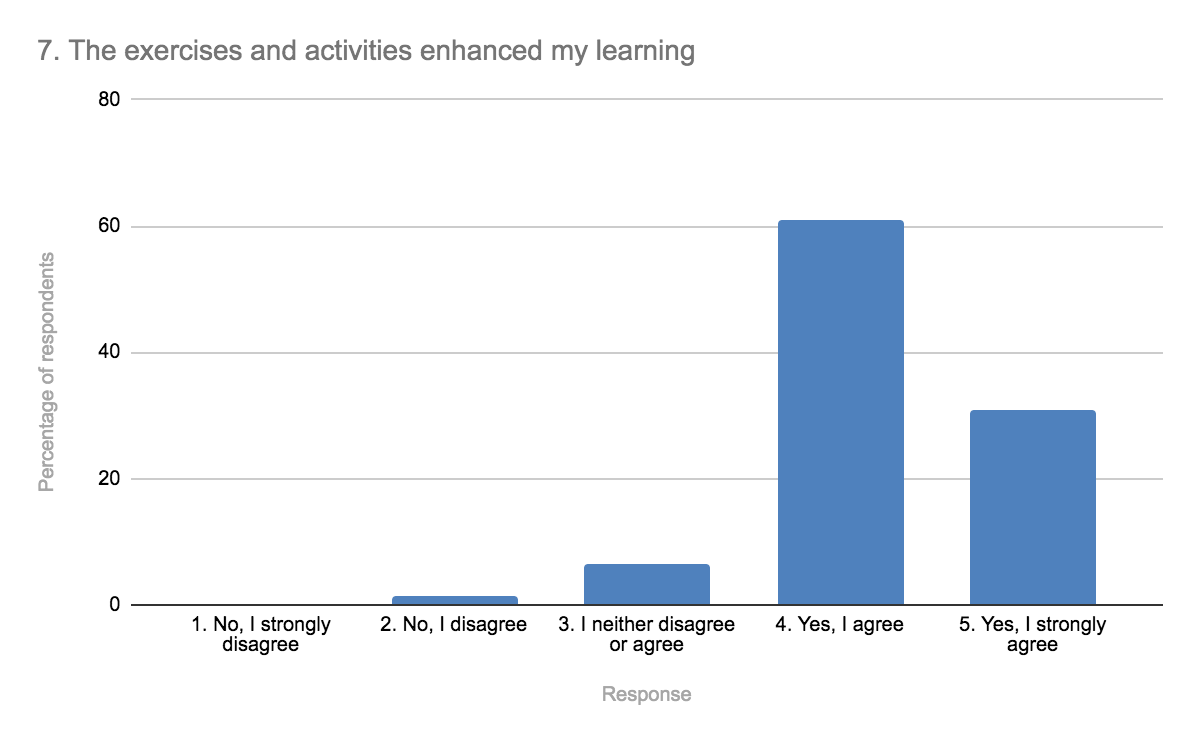


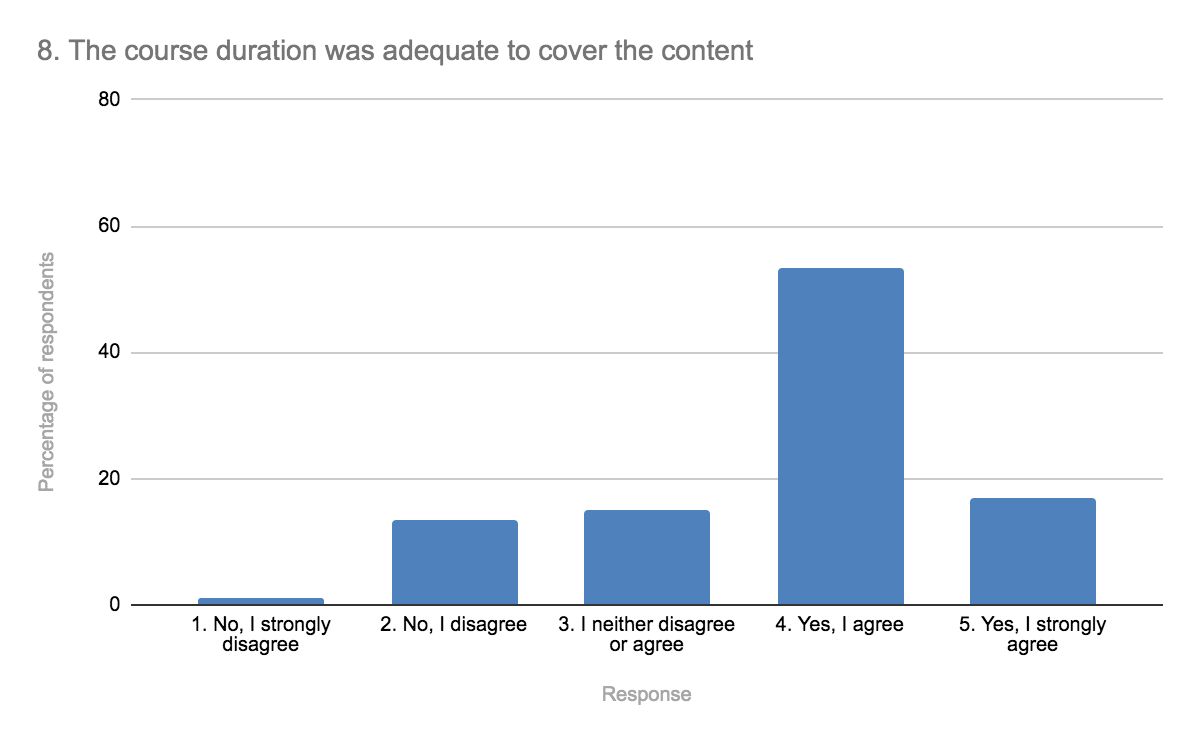


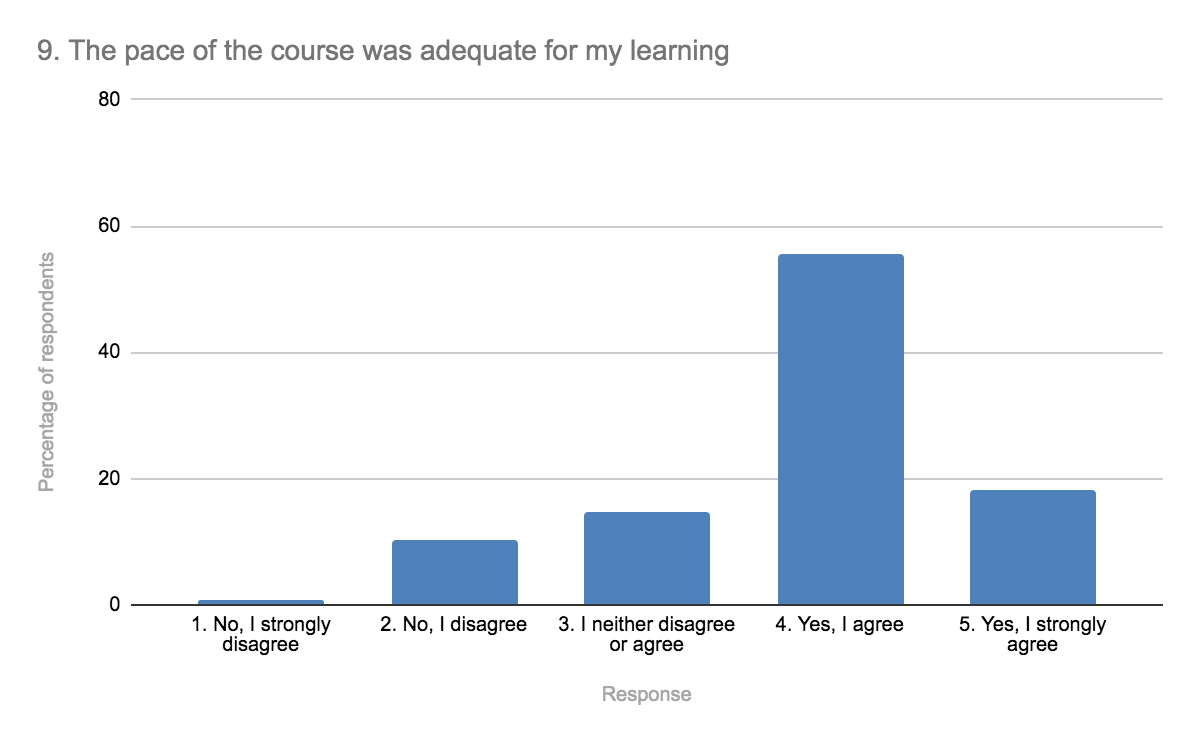


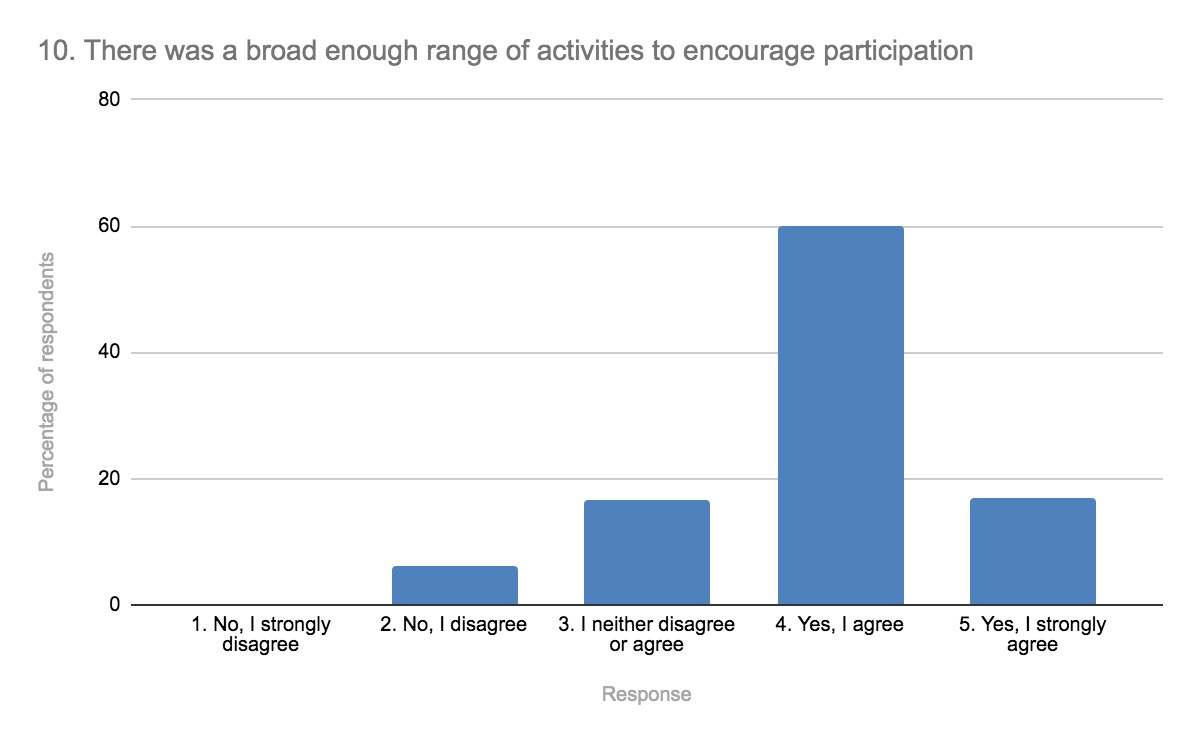


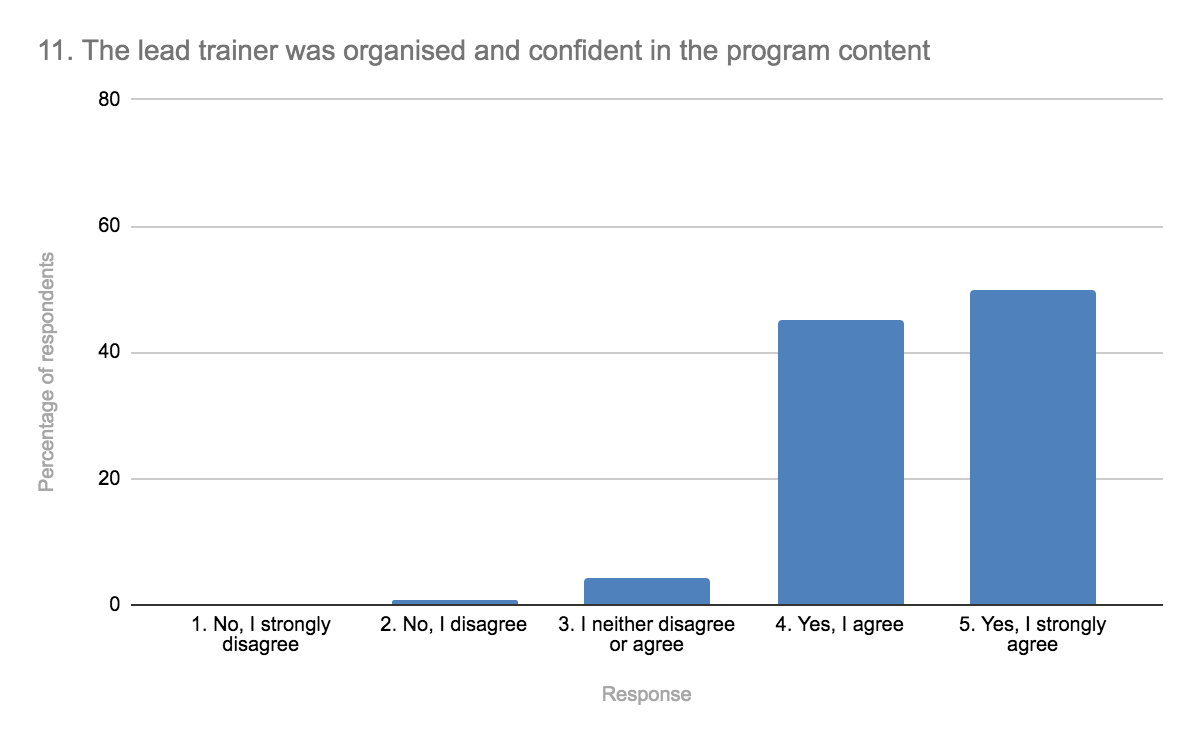


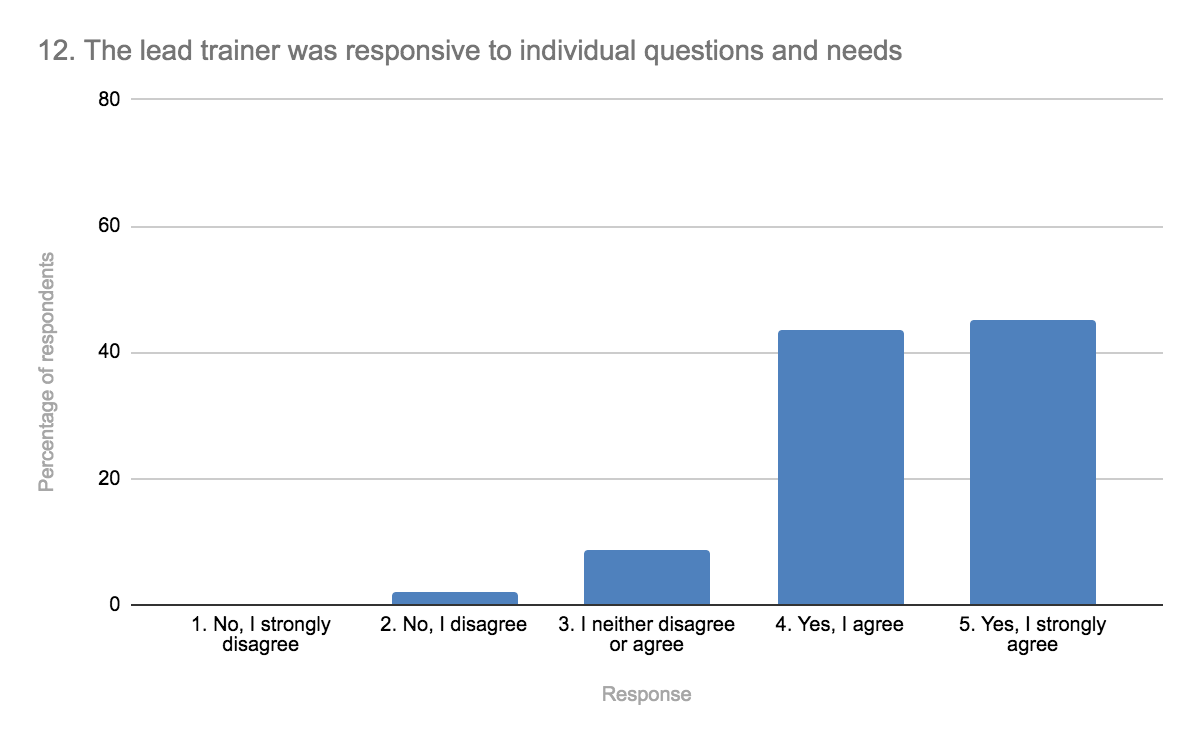


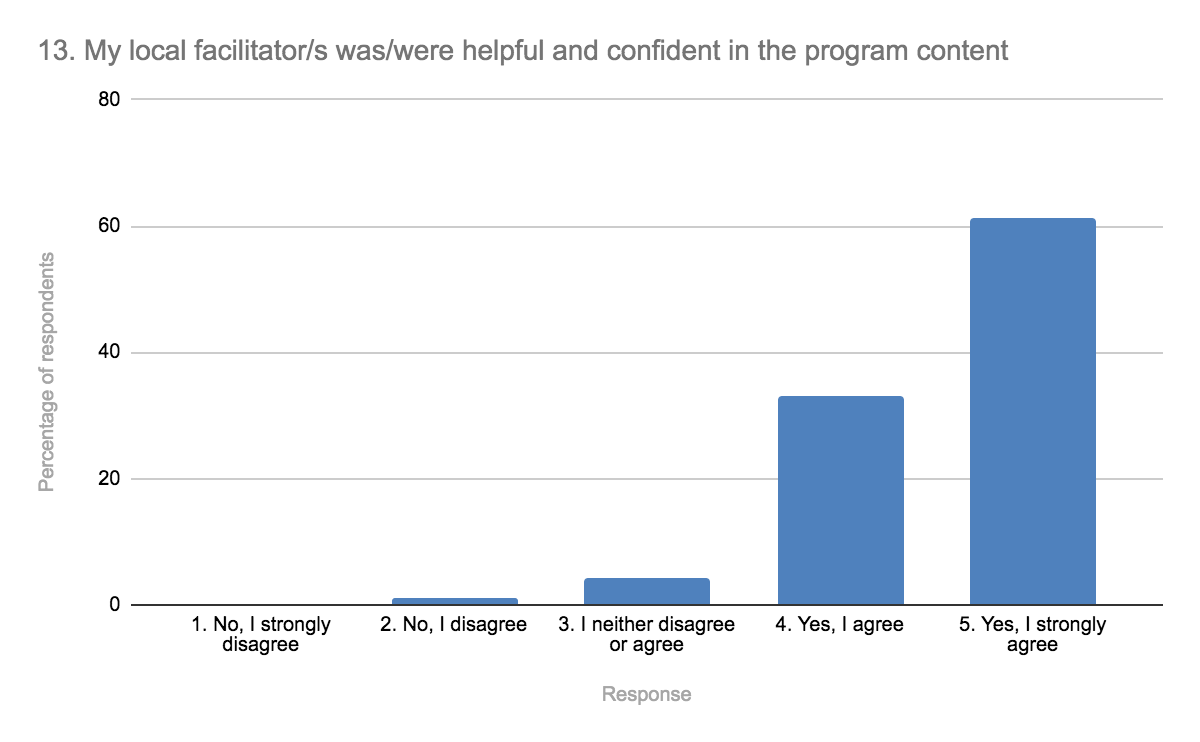


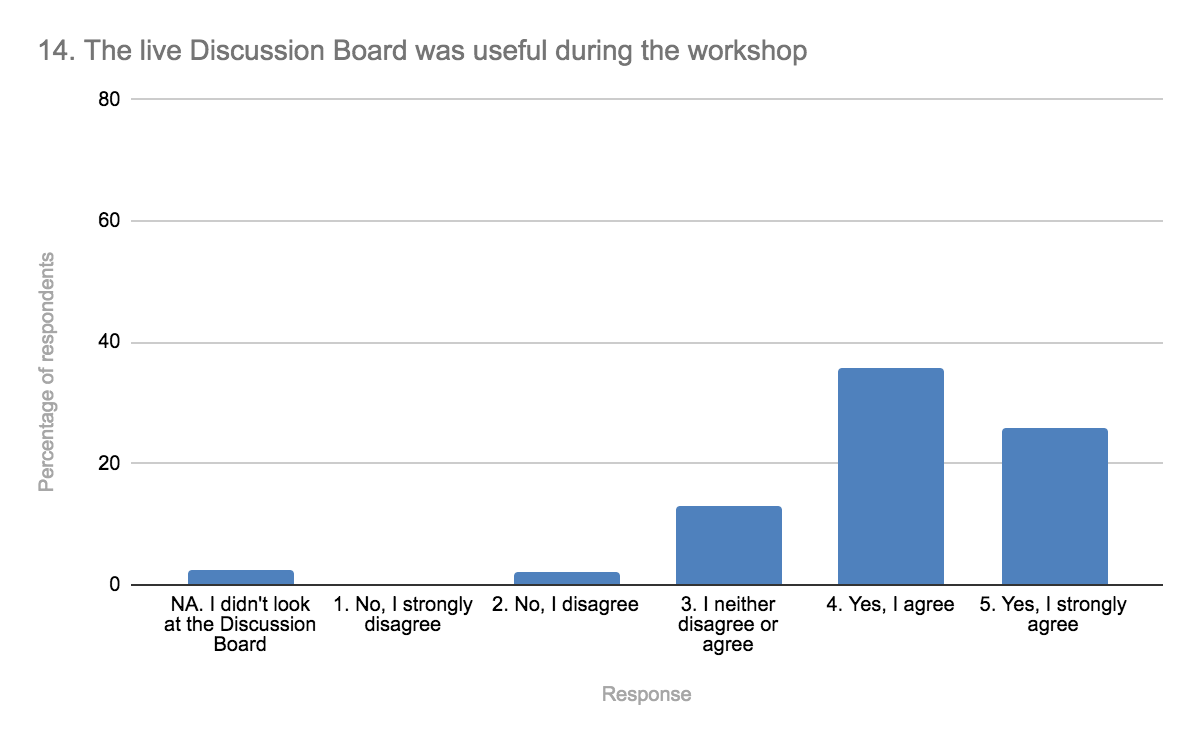

Supplement: S3 Table — (DOCX) [file pcbi.1008715.s004.docx]
